# A Comparison of HIV-Related Risk Factors Between Black Transgender Women and Black Men Who Have Sex with Men

**DOI:** 10.1089/trgh.2016.0003

**Published:** 2016-09-01

**Authors:** Elizabeth J. Siembida, Lisa A. Eaton, Jessica L. Maksut, Daniel D. Driffin, Robert Baldwin

**Affiliations:** ^1^ Department of Human Development and Family Studies, University of Connecticut, Storrs, Connecticut.; ^2^ SHARE Project, Atlanta, Georgia.

**Keywords:** HIV/AIDS, intersectionality, men who have sex with men, transgender

## Abstract

**Purpose:** Rates of HIV infection among transgender women (TW) are higher than rates observed among men who have sex with men (MSM), and black or African American individuals are at a disproportionately higher risk for HIV than individuals of other races. Limited information, however, is available regarding the needs of black TW and their risk for HIV. Numerous scholarly works and surveillance reports have combined TW with MSM, which has stymied our ability to understand the unique needs of black TW.

**Methods:** To identify patterns of HIV risk among black TW and black MSM, the current study utilized a cross-sectional, convenience sample to compare sociodemographic risk factors, HIV prevention tools, HIV-related risk factors, and psychosocial and sexual risk factors in a sample of 58 black TW and 764 black MSM. Participants were recruited between 2012 and 2014 from Atlanta, GA.

**Results:** Findings demonstrated that black TW were significantly more likely to report lower educational attainment (odds ratio [OR]=0.60, 95% confidence interval [CI]: 0.42–0.85, *p*=0.005), greater likelihood of being homeless (OR=2.49, 95% CI: 1.30–4.78, *p*=0.006), lower HIV testing knowledge (OR=0.66, 95% CI: 0.52–0.83, *p*=0.001), and higher likelihood of having engaged in transactional sex (OR=1.95, 95% CI: 0.99–3.83, *p*=0.052) compared to black MSM.

**Conclusions:** These findings highlight the need to understand how risk factors for HIV present themselves similarly and differently for both black TW and black MSM, and for HIV prevention programs and interventions to incorporate evidence-based content for each group.

## Introduction

Men who have sex with men (MSM) historically have been the sociodemographic group most heavily affected by the HIV epidemic in the United States.^1,2^ Recent research, however, suggests that rates of HIV infection among transgender women (TW) are higher than rates observed among MSM,^3,4^ yet few studies have focused exclusively on HIV risk in TW.^4^ Furthermore, research concerning both TW and MSM has found disproportionately higher rates of HIV infection among individuals who identify as black or African American.^1–3^ The relatively limited data on black TW and the high rates of HIV prevalence among this population suggest that there is an urgent need for further research with this population.^5–7^ Efforts to develop the literature on black TW, and TW more broadly, however, have been hindered by a common practice of including TW within samples of MSM.^5–7^ Although there is a growing literature on risk factors for HIV among TW, there remains a limited yet urgent need to focus on this population. For example, the CDC does not “uniformly collect” data on transgender populations.^8^ Based on the limited information regarding TW, a better understanding of their needs is warranted.

Overall, TW experience high rates of sociodemographic risk factors, including homelessness, transactional sex, lower educational attainment,^9–12^ high rates of substance abuse,^13,14^ and physical and sexual violence.^13–16^ Research focused on race/ethnicity and TW has found that black TW report an increased likelihood of drug use, engagement in transactional sex, and drug and alcohol use in the context of sex compared to other ethnic minority TW,^9,10,17^ and ethnic minority TW report lower educational attainment and increased engagement in condomless receptive anal intercourse compared to white TW.^12^

Despite previous research findings concerning black TWs increased risk for HIV compared to black MSM,^1–3^ few studies have compared patterns of HIV risk factors between black TW and black MSM. It is known, however, that minority identities are related to both health promotive and risk taking behavior. According to the intersectionality theoretical framework,^18–20^ multiple identities intersect at microlevels and influence experiences related to privilege and oppression at macrolevels. Intersectionality research highlights the notion that social categories are not independent, but “interdependent and multiconstitutive.”^21^ The focus on only one social category (or identity) does not comprehensively explain the disparate and unequal treatment faced by individuals of marginalized groups. Rather, intersectionality posits that unequal and disparate treatment can only begin to be understood when acknowledging the intersection of multiple identities. This framework provides an avenue through which the patterns of HIV risk factors can be contextualized among black TW and black MSM, and highlights the importance of this approach. According to the intersectionality framework, black TW and black MSM may experience risk factors for varying health outcomes differently, yet data from TW are frequently included with MSM or treated as a subcategory of MSM in HIV prevention work.

Consistent with the intersectionality framework, there is some evidence for differing health-related behaviors and sexual risk taking among black MSM and black TW.^22^ For example, race/ethnic minority TW engage in HIV risk taking behaviors (e.g., transactional sex) at higher rates than their race/ethnic minority MSM counterparts.^23^ On the whole, however, there exists limited research on why rates of HIV are higher for black TW compared with black MSM—with both groups experiencing alarmingly high rates of HIV. Sociodemographic variables, health protective behaviors, and internalized beliefs related to HIV prevention may vary across groups and affect rates of HIV among black TW and black MSM.^3,4^ Examining these categories of HIV risk and acknowledging multiple minority identities are critical for developing potential interventions relevant for black TW and black MSM.^24^

### Study objectives

Given the importance of understanding the needs of both black TW and black MSM, the primary objectives of the current study were to examine how HIV risk factors vary between black TW and black MSM. Specifically, we compared (1) sociodemographic variables (i.e., age, education, income, homelessness, sexual orientation, and employment), (2) HIV prevention tools (i.e., ever been tested for HIV, date of last HIV test, heard of pre-exposure prophylaxis (PrEP), taken PrEP, and would take PrEP), (3) HIV-related risk factors (i.e., HIV risk perception, condom use self-efficacy, HIV testing knowledge, and HIV status disclosure), and (4) psychosocial and sexual risk factors (i.e., depression, alcohol use before or during sex, drug use before or during sex, alcohol use, marijuana use, other drug use, condomless anal intercourse, and transactional sex) between black TW and black MSM to identify differences and similarities in patterns of HIV risk taking.

## Methods

### Sampling, recruitment, and enrollment

Participants for the current study were recruited in Atlanta, GA, from gay-identified bars, clubs, bathhouses, parks, and street locations; from online classifieds; and on social media (e.g., Facebook, Black Gay Chat, Jack'd) between January 2012 and March 2014. Participants were recruited for an ongoing longitudinal study examining HIV testing counseling interventions. The data presented here come from the baseline survey assessment. Individuals were eligible to participate if they reported engaging in condomless anal sex with a male partner in the past year, male or transgender female gender identity, HIV-negative or unknown status (individuals reporting HIV-positive status were referred to other available studies), and were at least 18 years of age. Before enrollment in the study, potential participants were screened to determine if they fit the study inclusion criteria. Participants recruited at LGBT-friendly locations were screened in-person when recruiters approached individuals as they entered the abovementioned venues, and participants recruited online were screened using telephone screening software.

Nine hundred fifty-nine participants screened eligible and attended an in-person appointment at the study research site in Atlanta, GA. During this appointment, participants provided consent and completed a survey assessment that utilized Audio Computer Assisted Interviewing (ACASI) software. Participants were compensated $30 for participating in the study. One hundred thirty-seven participants identified as a race/ethnicity other than black (i.e., white, Latino, Asian, other) and were, therefore, removed from all further analyses. Participants who identified as multirace, including black, were retained in analyses. The final sample for this study included 822 participants. All study procedures were approved by the University of Connecticut Institutional Review Board.

### Measures

#### Sociodemographic variables

Participants were asked to report on their age, years of education, employment status, income level, race/ethnicity, experiences with homelessness, and sexual orientation (i.e., whether they identified as same gender loving/gay, bisexual, or heterosexual). Our dependent variable, gender identity (i.e., male or transgender female), was established using the two-step method that relies on asking two questions to establish gender identity.^25,26^ Participants were first asked to report what gender they were born (i.e., male or female), and then, participants reported what gender they identify with (i.e., male, female, or transgender [male to female]). Participants were coded as male if they reported being born male and currently identifying as male, or were coded as transgender female if they reported being born male but identifying as female or identifying as transgender (male to female).

#### HIV prevention tools

##### PrEP knowledge and use

PrEP—or taking antiretrovirals (i.e., Truvada) before engaging in sex to prevent HIV infection^27^—was assessed to gauge the extent to which black TW and black MSM were aware of and using recent HIV prevention advances. Specifically, participants were asked whether they had heard of PrEP, had taken PrEP, and if they would like to take PrEP if provided the opportunity. For each question, participants answered *yes* or *no*.

##### HIV testing history

Participants' experience with HIV testing was assessed with two items. The first item asked if participants had ever been tested for HIV (dichotomous *yes* or *no* response), and the second item asked participants the date of their last HIV test (which was reported in number of months between date of assessment and date of test).

#### HIV-related risk factors

##### HIV risk perception

Participants were asked five questions^28^ regarding how much HIV risk they perceived under varying scenarios (e.g., “How risky is anal sex without a condom as the bottom partner with a man you just met who tells you his HIV status is negative?”). Responses ranged from 0=*no or low risk* to 10=*very high risk* and Cronbach's α=0.80. An average of the five items was calculated to create an HIV risk perception variable with higher scores indicating greater perceived HIV risk associated with condomless anal sex acts.

##### Condom use self-efficacy

To measure participants’ self-efficacy in negotiating condom use with a partner (e.g., “I feel confident in my ability to discuss condom usage with any partner I might have”), seven items were used from the Condom Use Self-Efficacy Scale (CUSES) by Brafford and Beck.^29^ These seven items were averaged to create a composite condom use self-efficacy variable, and higher scores represented greater condom use self-efficacy. Responses ranged from 1=*strongly disagree* to 6=*strongly agree* and Cronbach's α=0.90.

##### HIV testing knowledge

Participants were asked about their knowledge of HIV testing results. A total of five questions that have been used in previous research were provided to participants with *yes* or *no* response options (e.g., “It is possible to test HIV negative but really be HIV positive if someone is recently infected with HIV”).^30^ Participants were given a 1 for a correct answer and a 0 for an incorrect answer. Responses were summed with greater scores indicating higher HIV testing knowledge.

##### HIV status disclosure

By using three items adapted from Eaton et al.,^28^ the current study examined how confident participants were in their ability to discuss HIV status with a new sex partner (e.g., “I am certain that I can ask a new sex partner his HIV status.”). Participants responded on a Likert scale ranging from 1=*strongly disagree* to 6=*strongly agree* and Cronbach's α=0.77. An average of the three items was calculated, and greater scores indicated higher certainty in being able to discuss HIV status.

#### Psychosocial and sexual risk factors

##### Depressive symptoms

Participants completed the 10-item Center for Epidemiological Studies Short Depression Scale (CES-D 10), a screening questionnaire used to measure depressive symptoms (e.g., “In the past week, I was bothered by things that usually don't bother me”).^31^ Participants responded on a Likert scale ranging from 0=*less than 1 day* to 3=*5–7 days*, items were reverse coded, if necessary, and Cronbach's α=0.81. A sum was calculated to determine the total depression score, and a score of 10 or greater was used as the cutoff for indicating the need for further screening.^32^

##### Substance use history

Participants were asked two items related to substance use in the context of sex. Participants reported on the number of times they used alcohol before or during sexual activity in the past 3 months, and the number of times they used drugs before or during sexual activity in the past 3 months. Next, participants reported if they had consumed alcohol, marijuana, crack/cocaine, nitrate inhalants, methamphetamines, erectile dysfunction drugs (without a prescription), injected drugs, or other drugs in the past 3 months. Three variables were created from these items: (1) alcohol use, (2) marijuana use, and (3) other drug use (all remaining drugs were combined due to low frequencies). Responses included a dichotomous *yes* or *no*.

##### Sex behaviors and sexual risk taking

Participants were asked to report the total number of times they engaged in condomless anal intercourse with a man in the past 3 months. Transactional sex was also assessed by asking participants if they had ever received money, food, a place to stay, or alcohol/drugs in exchange for sex. Responses included a dichotomous *yes* or *no*. Responses were summed across each item, and individuals were given a score of 0 if they had never received any resource in exchange for sex, and a score of 1 if they had received at least one resource in exchange for sex.

### Data analysis

Means, standard deviations, *N*s, and percentages were provided for each group on all variables of interest. Bivariate tests, including independent samples *t*-test with pooled variances, Mann–Whitney *U* test, or chi-square (*χ*^2^), were conducted, as appropriate (Tables 1 and 2). Generalized linear modeling was used to conduct multivariable logistic regression to assess the potential differing patterns of risk factors for HIV between black TW and black MSM (Table 3). Generalized linear modeling was utilized because of its flexibility in allowing for non-normal distributions among the included variables.

**Table 1.** **Sociodemographic Factors Among Black Transgender Women and Black Men Who Have Sex with Men Residing in the Atlanta Metro and Surrounding Areas**

	BTW (*N*=58)		BMSM (*N*=764)				
	*M*	*SD*	*M*	*SD*	*t*-test	df	*p*
Age	38.57	13.41	33.13	11.14	−3.01	63.12	0.004
	*n*	%	*n*	%	*χ*^2^		
Education							
Less than high school	8	13.79	40	5.24	34.30	5	<0.001
High school	34	58.62	227	29.71			
Some college	13	22.41	313	40.97			
College degree	2	3.45	133	17.41			
Graduate school	1	1.72	31	4.06			
Graduate degree	0	0.00	20	2.62			
Income							
$0–10,000	46	79.31	399	52.23	20.06	6	0.003
$11,000–20,000	5	8.62	144	18.85			
$21,000–30,000	2	3.45	104	13.61			
$31,000–40,000	2	3.45	52	6.81			
$41,000–50,000	0	0.00	33	4.32			
$51,000–60,000	0	0.00	18	2.36			
$61,000 or higher	1	1.72	10	1.31			
Homelessness	32	55.17	172	22.51	32.12	1	<0.001
Sexual orientation							
Gay/homosexual/same gender loving	20	34.48	358	46.86	3.49	2	0.175
Bisexual	24	41.38	302	39.53			
Straight/heterosexual^a^	11	18.97	97	12.70			
Employment status							
Unemployed	49	84.48	473	61.91	13.25	1	<0.001
Employed	8	13.79	291	38.09			

^a^ Men who identified as straight/heterosexual were included in the analysis if they reported at least one instance of engaging in male-to-male sexual contact (*N*=1 participant was excluded for identifying as straight/heterosexual and reporting no male-to-male sexual contact).

BMSM, black men who have sex with men; BTW, black transgender women.

**Table 2.** **HIV-Related, Psychosocial, and Sexual Risk Factors Among Black Transgender Women and Black Men Who Have Sex with Men**

	BTW (*N*=58)		BMSM (*N*=764)				
	*M*	*SD*	*M*	*SD*	Mann–Whitney *U* test	df	*p*
HIV prevention tools							
Months since last HIV test^a^	20.21	34.12	14.71	28.71	−1.69		0.091

	*n*	%	*n*	%	*χ*^2^		
Ever been tested for HIV	42	72.41	636	83.25	3.46	1	0.063
Heard of PrEP	10	17.24	184	24.08	1.26	1	0.262
Taken PrEP	1	1.72	12	1.57	0.18	1	0.668
Would take PrEP	39	67.24	624	81.68	6.49	1	0.011

	*M*	*SD*	*M*	*SD*	*t*-test		
HIV-related risk factors							
HIV testing knowledge	2.86	1.16	3.77	1.20	5.55	819	<0.001

	*M*	*SD*	*M*	*SD*	Mann–Whitney *U* test		
Condom use self-efficacy	5.04	1.17	5.21	1.07	−0.67		0.500
HIV risk perceptions	7.06	2.08	7.47	1.66	−0.94		0.357
HIV status disclosure	4.45	1.60	5.10	1.21	−3.02		0.003

	*M*	*SD*	*M*	*SD*	*t*-test		
Psychosocial and sexual risk factors							
Depressive symptoms (CES-D)	10.77	6.12	10.11	6.31	−0.76	819	.446

	*M*	*SD*	*M*	*SD*	Mann–Whitney *U* test		
Condomless anal intercourse	4.93	9.06	4.86	8.46	−0.63		0.527
Number of times alcohol used before or during sex in past 3 months	5.47	10.22	5.61	10.43	−0.09		0.928
Number of times drugs used before or during sex in past 3 months	5.93	13.12	5.43	13.57	−0.40		0.404

	*n*	%	*n*	%	*χ*^2^		
Engaged in transactional sex	40	68.97	334	43.71	14.83	1	<0.001
Used alcohol in past 3 months	44	75.86	688	90.05	9.58	1	0.002
Used marijuana in past 3 months	37	63.79	416	54.45	2.29	1	0.131
Used drugs (except marijuana) in past 3 months	31	53.45	284	37.17	6.65	1	0.010

^a^ Within the study sample, 128 individuals (15.6%) said they never tested for HIV and therefore this variable could not be calculated for these individuals. Due to this fact, this variable was eliminated from the multivariable model.

CES-D, Center for Epidemiological Studies Short Depression Scale; PrEP, pre-exposure prophylaxis. BMSM, black men who have sex with men; BTW, black transgender women.

**Table 3.** **Odds Ratios and 95% Confidence Intervals from the Multivariable Logistic Regression Model with Gender Identity as the Dependent Variable**

Independent variables	OR	95% CI	*p*
Age	1.01	(0.98–1.03)	0.631
Education	**0.60**	**(0.42–0.85)**	**0.005**
Income	0.93	(0.67–1.30)	0.681
Homelessness	**2.49**	**(1.30–4.78)**	**0.006**
Employment status	1.67	(0.69–4.02)	0.252
Ever been tested for HIV	0.87	(0.44–1.71)	0.688
Would take PrEP	0.53	(0.27–1.02)	0.058
HIV testing knowledge	**0.66**	**(0.52–0.83)**	**0.001**
HIV status disclosure	0.91	(0.75–1.11)	0.351
Engaged in transactional sex	**1.95**	**(0.99–3.83)**	**0.052**
Used alcohol in last 3 months	**0.33**	**(0.16–0.70)**	**0.004**
Used drugs (except pot) in last 3 months	0.94	(0.48–1.85)	0.855

Variables found to be significantly associated with gender identity are bolded.

Black MSM were used as the reference category in the multivariable logistic regression analysis.

CI, confidence interval; OR, odds ratio.

The following independent variables were analyzed in bivariate analyses with gender identity (black TW or black MSM) as the dependent variable: (1) *sociodemographic variables*—age, education, income, homelessness, sexual orientation, and employment, (2) *HIV prevention tools*—ever been tested for HIV, date of last HIV test, heard of PrEP, taken PrEP, and would take PrEP, (3) *HIV-related risk factors—*HIV risk perceptions, condom use self-efficacy, HIV testing knowledge, and HIV status disclosure, and (4) *psychosocial and sexual risk factors*—depression, alcohol use before or during sex, drug use before or during sex, alcohol use, marijuana use, other drug use, condomless anal intercourse, and transactional sex.

Independent variables in the bivariate analyses that were found to be significantly different between black TW and black MSM at the *p*<0.10 level were included in the multivariable analysis. In the multivariable analysis, variables were entered simultaneously and odds ratios (ORs) and their 95% confidence intervals (CIs) were calculated. For the multivariable analysis, *p*<0.05 was used to define statistical significance. Data analyses were completed between June 2015 and August 2015, and less than 5% of data were missing for any given variable. We used PASW Statistics, version 19.0 (SPSS, Inc., IBM, Somers, NY) for all analyses.

## Results

### Sociodemographic variables

Of the total sample, 58 participants (7%) identified as black TW and 764 (93%) identified as black MSM. Black TW were significantly more likely to be older [*t*(63.12)=−3.01, *p*=0.004], less educated [*χ*^2^(5)=34.30, *p*<0.001], unemployed [*χ*^2^(1)=13.25, *p*<0.001], have lower incomes [*χ*^2^(6)=20.06, *p*=0.003], and experience homelessness [*χ*^2^(1)=32.12, *p*<0.001] compared with black MSM (Table 1).

### HIV prevention tools

Overall, 678 respondents (82.5%) reported having been tested for HIV at least once in their lifetime. Bivariate analyses found that black TW were less likely to have been tested for HIV [*χ*^2^(1)=3.46, *p*=0.063], and for those participants who reported having an HIV test, more time had passed since black TW had an HIV test compared to black MSM (Mann–Whitney *U*=−1.69, *p*=0.091). Although there were few differences between black TW and black MSM on their knowledge and use of PrEP, black TW were significantly less likely to want to use PrEP if they had access to it compared with black MSM [*χ*^2^(1)=6.49, *p*=0.011] (Table 2).

### HIV-related risk factors

On average, participants reported an HIV risk perception score of 7.45 (*SD*=1.70), indicating medium to high levels of risk, and there was no statistically significant difference on HIV risk perception between black TW and black MSM. The mean condom use self-efficacy score for the total sample was 5.20 (*SD*=1.08), and there was no statistically significant difference on condom use self-efficacy between black TW and black MSM. Black TW were significantly less likely to feel comfortable having discussions about HIV status with a new partner (Mann–Whitney *U*=−3.02, *p*=0.003) compared with black MSM. When asked about HIV testing knowledge, black TW scored significantly lower, indicating less understanding about HIV testing and HIV test results than black MSM [*t*(819)=5.55, *p*<0.001] (Table 2).

### Psychosocial and sexual risk factors

Overall, 389 (47.3%) participants scored 10 and above on the CES-D 10, the threshold score indicating the need for further screening for depression. There were no significant differences on CES-D 10 scores across groups. Participants reported drinking alcohol before or during sex 5.60 times (*SD*=10.41) in a 3-month period, and using drugs before or during sex 5.46 times (*SD*=13.52) in a 3-month period. Seventy-six percent of black TW and 90.1% of black MSM reported drinking alcohol in the past 3 months, 63.8% of black TW and 54.5% of black MSM reported using marijuana in the past 3 months, and 53.5% of black TW and 37.2% of black MSM reported using any drugs (except marijuana) in the past 3 months. Black TW were significantly more likely to report using any drug (except marijuana) in the last 3 months [*χ*^2^(1)=6.65, *p*=0.010] and significantly less likely to report drinking alcohol in the last 3 months [*χ*^2^(1)=9.58, *p*=0.002] compared with black MSM.

Although all participants in the study reported at least one act of condomless anal intercourse in the last year as part of the study's inclusion criteria, there was no significant difference in the total number of condomless anal intercourse acts reported by black TW and black MSM. Sixty-eight percent of black TW reported ever engaging in transactional sex versus 43.7% of black MSM [*χ*^2^(1)=14.83, *p*<0.001] (Table 2).

### Multivariable logistic regression model

After identifying HIV risk factors in the bivariate analysis that were associated with gender identity at the *p*<0.10 level, these risk factors were examined in a multivariable model. Multiple variables were found to be significantly associated with gender identity. Specifically, in the multivariable model, lower educational attainment (OR=0.60, 95% CI: 0.42–0.85, *p*=0.005), higher likelihood of being homeless (OR=2.49, 95% CI: 1.30–4.78, *p*=0.006), lower understanding of HIV test results (OR=0.66, 95% CI: 0.52–0.83, *p*=0.001), and higher likelihood of having engaged in transactional sex (OR=1.95, 95% CI: 0.99–3.83, *p*=0.052) were associated with identifying as black TW. Black MSM were more likely to have consumed alcohol in the last 3 months (OR=0.33, 95% CI: 0.16–0.70, *p*=0.004) than black TW.

## Discussion

Overall, the extent to which black TW and black MSM experience disadvantage and, more specifically, risk factors associated with HIV transmission, is alarming. Both black TW and black MSM reported sociodemographic factors and behaviors associated with increased risk for HIV in the current study, but varying patterns of HIV risk were found among these two populations. It is important to consider these findings in the context of the intersectionality framework, as the greater psychosocial-related burdens observed among TW in the current study highlight the need to acknowledge and understand the intersection of the multiple minority identities. Specifically, this approach is important because it can be used to guide how outreach and linkage to care programs (and other related engagement programs) are shaped for addressing the unique needs of each population.^18–20^ For example, TW may experience uncertainty in linking to HIV prevention care when much of the programming is targeted to MSM. Without acknowledging multiple identities, there is the potential for overlooking factors that are critical to the success of improving the social conditions of each minority group. Furthermore, the findings are consistent with previous research focused on TW,^33^ and this study is among the first to compare the patterns of HIV risk factors between black TW and black MSM.

Although data from the current study suggest that both black MSM and black TW face numerous sociodemographic- and psychosocial-related burdens, black TW reported lower education attainment and higher rates of homelessness and engagement in transactional sex. Given what is understood about the relationships between sociodemographic risk factors for HIV and actual HIV transmission rates,^34–36^ these disparities place black TW at greater risk for HIV transmission. Homelessness, in particular, is a notable factor for increasing likelihood of HIV transmission as this relationship is thought to exist through the reliance on transactional sex for getting basic needs met (e.g., shelter). As a marginalized group with intersecting minority identities, difficulty in securing employment (e.g., via discrimination, prejudice, and bias against gender minority populations), and, therefore, experiencing an inability to gain basic living necessities are likely important drivers in their engagement in transactional sex compared to black MSM.^37,38^

The stigma related to homelessness may also act as a social barrier to seeking out healthcare, and, potentially, to exposure to sexual networks where HIV prevalence is elevated.^39,40^ There exist, however, very limited data on these disparities specifically related to TW. Prior work among race minority TW has found that experiencing structural barriers, including homelessness, has been associated with a greater likelihood of experiencing transphobic victimization (emotional, physical, and sexual abuse due to being transgender) and engaging in sexual risk taking.^41^ Research with black MSM has also found that unstable housing is associated with condomless sex with a serodiscordant partner.^42^ In the same sample of black MSM, lower educational attainment was associated with condomless sex with a nonmain sex partner.^42^ On the whole, establishing a comprehensive understanding of multilevel factors related to HIV transmission for both black TW and MSM populations is imperative for HIV prevention and treatment, and the accumulation of these factors likely places black TW at higher risk for HIV infection.^43^

The lack of access to basic living necessities also impacts black TWs linkage to healthcare and HIV prevention knowledge.^44,45^ With lower rates of accessing HIV test results and lowered interest in using prevention tools such as PrEP, black TW remain at an increased risk for HIV compared to black MSM. Facilitating access to HIV testing and counseling is an urgent first step in reducing the burden of HIV among black TW. Attaining this goal, however, is reliant on HIV prevention programs, including engagement strategies and intervention components focused on the unique needs of black TW.^46^

The high rate of depressive symptoms reported among both black TW and black MSM in the current study is noteworthy. Reisner et al.^47^ highlight the relationship between depression and increased risk for HIV among black MSM, and the need to address screening and treatment for depression before engagement in further intervention programs. Slightly less than half of the sample reported depressive symptoms that indicate the need for further screening. Examining this finding in combination with the previous findings regarding sociodemographic risk factors among black TW and black MSM reinforces the urgency of meeting the basic needs of black TW and black MSM. Failing to meet these needs likely prohibits the implementation of recent advances in prevention options (e.g., PrEP).

### Limitations

The findings from the current study should be interpreted in light of their limitations. Specifically, the data presented in this study were collected with a cross-sectional approach, and therefore, any conclusions based on causality or the directions of specific relationships cannot be made. In addition, the participants in the current study were recruited from LGBT-friendly venues or through online venues targeted toward LGBT populations, and participants reported condomless anal sex in the past year as part of eligibility screening. The use of these sampling techniques may bias the conclusions of the study. Specifically, the study does not include black TW who only have sex with women, individuals who do not engage in condomless anal sex, or black TW and black MSM who frequent other venues. The lack of representation of these individuals in the current study may underrepresent HIV risk factors among all black TW and black MSM, and likely affects our observed patterns of risk taking. Finally, all of the participants in the current study lived in the Atlanta metropolitan and surrounding areas, and their experiences may not generalize to other regions or rural populations of black TW and black MSM.

## Conclusion

The current study highlights the need to consider black TW separate from black MSM when examining HIV risk in these populations. Likewise, when creating HIV prevention programs, researchers/community agencies need to carefully consider the audience they are targeting and evaluate the needs of programming specific to their target population. As posited by intersectionality theory, the multiple minority statuses of black TW must be given strong consideration when developing outreach efforts and intervention content—doing so will ensure that HIV prevention and treatment programs appeal to and reach the needs of black TW.

## Abbreviations Used

ACASI: Audio Computer Assisted Interviewing
BMSM: black men who have sex with men
BTW: black transgender women
CES-D 10: Center for Epidemiological Studies Short Depression Scale
CIs: confidence intervals
CUSES: Condom Use Self-Efficacy Scale
MSM: men who have sex with men
ORs: odds ratios
PrEP: pre-exposure prophylaxis
TW: transgender women
